# Prevalence and risk factors for chronic kidney disease in Indonesia: An analysis of the National Basic Health Survey 2018

**DOI:** 10.7189/jogh.12.04074

**Published:** 2022-10-14

**Authors:** Ni Made Hustrini, Endang Susalit, Joris I Rotmans

**Affiliations:** 1 Division of Nephrology and Hypertension – Department of Internal Medicine, Faculty of Medicine – Universitas Indonesia, Dr. Cipto Mangunkusumo National General Hospital – Jakarta, Indonesia; 2 Department of Internal Medicine, Leiden University Medical Center, Leiden, the Netherlands

## Abstract

**Background:**

The prevalence of chronic kidney disease (CKD) in Indonesia is rising, but the exact extent of the burden of CKD in Indonesia is unknown. To design a screening program for individuals at high-risk, more knowledge is required regarding the prevalence and risk factors of CKD in Indonesia. The latter could have a big impact on the prevention and management of patients with CKD in Indonesia.

**Methods:**

For this purpose, we analysed data from The National Basic Health Survey 2018 (Riset Kesehatan Dasar, Riskesdas 2018), a descriptive cross-sectional study in 34 provinces, 416 districts and 98 cities in Indonesia. We included subjects aged ≥18 years and analysed the prevalence of CKD. Using multiple logistic regression, we investigated the association between CKD and potential risk factors such as demographic factors (age, gender, occupational status, level of education), lifestyle and behaviours (consumption of salty food, consumption of fruit and vegetables, smoking, alcohol consumption, carbonated drink consumption, physical activity), comorbid conditions (hypertension, heart disease, diabetes mellitus, hepatitis, stroke, nutritional status) and others (clean water supply, pregnancy complication, access to health care).

**Results:**

We included 389 093 subjects in this study out of 713 783 subjects that participated in Riskesdas 2018 survey. The prevalence of CKD was 0.5%. The survey included mostly younger adults age 18-59 years (83.1%) with a mean (SD) age of 44.3 (15.1) years. The majority of subjects were female (60.3%), unemployed (58.4%), and the proportion of obese subject was 25.4%. Hypertension was the major comorbid condition (40.8%), while the proportion of diabetes mellitus (DM), heart disease, stroke and hepatitis were quite low (3.3%, 2.6%, 1.7% and 0.5%; respectively). Despite the high proportion of hypertension, only 36.2% of subjects did receive a prescription for anti-hypertensive medication of which only 21.7% used this medication regularly. The multiple logistic regression analysis demonstrated that hepatitis was the strongest risk factor of CKD (odds ratio (OR) = 3.406; 95% confidence interval (CI) = 2.496-4.648), exceeding the risk of CKD in patients with physical inactivity (OR = 1.236; 95% CI = 1.128-1.354), low education status (OR = 1.307; 95% CI = 1.191-1.434), male (OR = 1.527; 95% CI = 1.398-1.668), stroke (OR = 1.916; 95% CI = 1.570-2.338), heart disease (OR = 2.941; 95% CI = 2.356-3.671), and DM (OR = 2.462; 95% CI = 1.979-3.063). We also observed that DM (OR = 4.280; 95% CI = 3.756-4.876) and male subjects (OR = 1.474; 95% CI = 1.352-1.606) were identified as independent risk factors for CKD in hepatitis-positive subjects.

**Conclusions:**

This population-based survey confirmed the increasing burden of CKD in Indonesia and suggested that besides traditional metabolic risk factors, viral hepatitis has proven to be an independent risk factor for CKD in Indonesia. Furthermore, the risk of CKD is greater in male hepatitis patients with DM. The result of this study demonstrates the need for an aggressive screening program for patients with a high risk for the development of CKD. Apart from patients with traditional cardiometabolic risk factors, such a program should include patients with viral hepatitis.

Chronic kidney disease (CKD) is a major global health issue. Obviously, the incidence and prevalence vary substantially between countries due to differences in underlying diseases rates and availability of medical treatment options [1]. The incidence of CKD reached 200 cases per million per year in many countries, although prevalence varies between countries: eg, US, Taiwan and certain regions in Mexico are approaching almost 400 cases per million [1-3]. In 2017 the global prevalence of CKD was 9.1%, ranging from 8.5% to 9.8%, while a third of CKD patients lived in China and India [2]. In the US, the CKD prevalence was around 11.5% (1996 to 2006) [1].

Like other parts of the world, Indonesia equally suffers from a high burden of CKD. Nevertheless, data regarding the epidemiology of CKD in Indonesia is scarce and inconsistent. The National Basic Health Research (Riset Kesehatan Dasar, Riskesdas), reported that the CKD (eGFR<60 ml/min/1.73 m^2^) prevalence was 3.8 permil (‰) in 2018, increased from 2.0 permil (‰) in 2013 [4]. However, these data may underestimate the real number of CKD patients as screening for CKD is notoriously challenging [5]. Meanwhile, Prodjosudjadi and coworkers found the prevalence of CKD was 12.5% of subjects with either hypertension, proteinuria, and/or diabetes mellitus (DM) [6]. This data fits well with international studies on CKD prevalence and burden of the disease [1-3].

Besides the incidence and prevalence of CKD in Indonesia, the aetiology of CKD in Indonesia is not well recorded either. Indonesia is still facing the triple burden of disease. First is as a result of the ineffective control of infectious, re-emerging, and newly emerging diseases; second is due to the rise of chronic diseases into the top five list of catastrophic disorders as a result of demographic and nutritional transitions; and lastly because of the steady increase in the incidence of trauma and injuries [7]. In addition, the influence of environmental factors also needs to be considered in the progression of CKD, especially the clean water source which could contribute to the waterborne diseases and diarrheal illnesses leading to acute kidney injury (AKI), schistosomiasis that can cause CKD, and water pollution that further causing CKD [8,9].

The unique characteristics of the Indonesian population has raised question into the extent to which this heterogeneity will impact the aetiology of CKD. Moreover, these data might point toward the necessity to look for the specific causative factors for CKD in our population. The question arises whether it has changed in recent years and how significant it will influence the strategy to overcome the burden of CKD in our population. Therefore, in this study we tried to analyse the risk factors of CKD in Indonesian population using the data from our most recent national survey from 2018.

## METHODS

The National Basic Health Research (Riset Kesehatan Dasar – Riskesdas) was a descriptive cross-sectional survey held by The Indonesian Ministry of Health since 2007 to collect basic data and health indicators of Indonesian citizens which represents the national, provincial and district/city region. The Riskesdas survey conducted in March 2018 covers all provinces and districts/cities (34 provinces, 416 districts and 98 cities) in Indonesia.

### Selection and description of participants

We included subjects age ≥18 years for further analysis of the prevalence of CKD and relation to any risk factors of CKD such as demographics (age, gender, occupation status, level of education), behaviour and life style (consumption of salty food, consumption of fruit and vegetables, smoking, alcohol consumption, carbonated drink consumption, physical activity), comorbid condition (hypertensive, heart disease, diabetes, hepatitis, stroke, nutrition status) and others (clean water supply, pregnancy complication, access to health care facility). The Riskesdas survey included 713 783 subjects over 15 years old, which we filtered and adjusted to our inclusion criteria (subjects age <18 years old: n = 55 582). All subjects with missing data on studied variables were excluded from the analysis. Missing data in each stage of data cleaning included data on diagnosis of hypertension by physician (n = 266 628), and an extreme body mass index (BMI) (ie, BMI<14 kg/m^2^ (n = 877) and >51 kg/m^2^ (n = 164)). Initial missing data on body weight (n = 4240), height (n = 5046), first blood pressure measurement (n = 2020) and second blood pressure measurement (n = 10 997) were included using imputation strategy after we ensure that the data were missing at random.

### Study outcomes

The primary outcome of this study was to evaluate the prevalence of CKD and risk factors associated with CKD in Indonesia.

The secondary outcome of this study was to analyse the risk factors for CKD in Indonesia, i.e demographics (age, gender, occupation status, level of education), behaviour and life style (consumption of salty food, consumption of fruit and vegetables, smoking, alcohol consumption, carbonated drink consumption, physical activity), comorbidities (hypertensive, heart disease, diabetes, hepatitis, stroke, nutrition status) and others (clean water supply, pregnancy complication, access to health care facility).

### Case definition

The case definition applied in this study referred to the case ascertainment employed in the National Basic Health Survey (Riskesdas) 2018 data set [4].

Chronic kidney disease defined when a subject has been diagnosed with chronic kidney disease (within the last 3 months) by a physician.

Diabetes mellitus defined when a subject was previously diagnosed with DM by a physician.

Heart disease was any form of heart disease including congenital heart disease diagnosed by a physician previously.

Hypertension defined as measurement of blood pressure based on JNC VII criteria (systolic blood pressure >140 mm Hg and/or diastolic blood pressure >90 mm Hg), and previous diagnosis of hypertension made by a physician.

Stroke defined when the subject has been diagnosed with a stroke by a health care worker (doctor/nurse/midwife), or has never been diagnosed with stroke but has experienced sudden paralysis on one side of the body, paralysis on one side of the body accompanied by tingling, numbness, slanted mouth without paralysis of the eye muscles, slurred speech or difficulty speaking/communicating and/or unable to communicate.

Hepatitis defined when a subject was diagnosed with hepatitis previously by a physician based on blood examination.

Data collection on the frequency of physical activity was reviewed in the last week for subjects aged >10 years. Strenuous physical activity was defined as an activity continuously performed for at least 10 minutes until the pulse and breathing rate increases faster than usual (eg, drawing water, climbing mountains, running fast, cutting trees, hoeing, etc.) for at least three days a week with the total activity time >1500 MET minute. MET minute of strenuous physical activity was the length of time (minutes) of doing activity in one week multiplied by 8 calories. Moderate physical activity if doing moderate physical activity (sweeping, mopping, etc.) for at least five days or more with a total length of activity of 150 minutes in one week. Activity outside these two conditions is included in light physical activity (WHO GPAQ, 2012; WHO STEPS, 2012). The criteria for “active physical activity” were individuals who did strenuous or moderate physical activity, or both, while the “less active” criteria were individuals who did not do moderate or strenuous physical activity.

Information on the frequency and portion of vegetable and fruit intake was collected by calculating the number of days of consumption in a week and the average number of servings in a day. Subjects were categorized as ‘adequate' to consume vegetables and/or fruit if they ate at least 5 portions of vegetables and/or fruit per day for 7 days a week. Subjects were categorized as 'less' if the consumption of vegetables and/or fruit was less than the above provisions.

### Statistics

Statistical analysis was performed with SPSS version 20. The characteristics of the subjects were examined using univariate analysis for all variables. We further studied the proportion of difference between subjects with and without CKD in bivariate analysis using the χ^2^ statistic. A *P*-value of <0.05 was considered statistically significant. The association between CKD and risk factors were examined using multiple logistic regression models: first step was variables selection from bivariate model when *P*-value <0.25. The next step was adjustment of potential confounding factors along with the interaction test to examine the interaction between the risk factors variables of the development of CKD. Finally, we performed multiple logistic regression analysis to determine the independent risk factors for CKD.

## RESULTS

### Description of study participants

**Figure 1:**
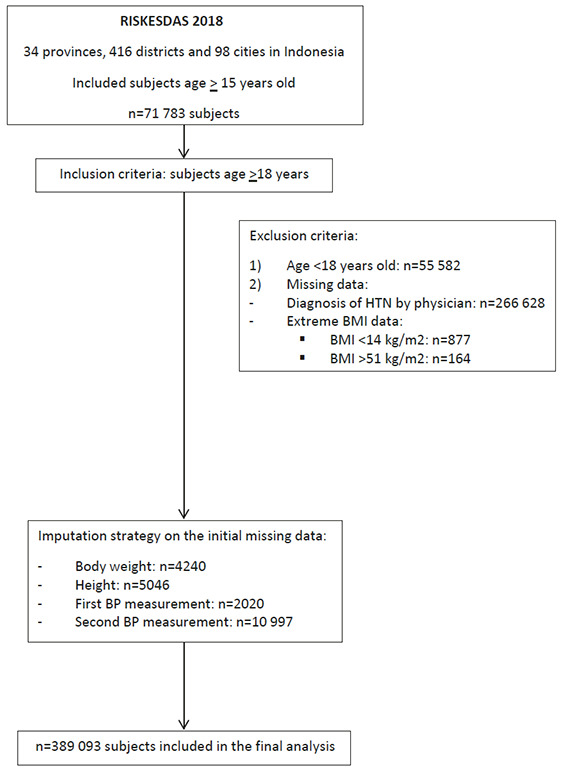
Flowchart of subjects’ recruitment.

A total of 389 093 subjects were included in this study (**Figure 1**). The mean (SD) age of the subjects was 44.3 (15.1) years with the highest proportion in the 18-59 years age group (83.1%). The survey included mostly female (60.3%) subjects, that were unemployed (58.4%) whereas the level of education was mostly mid to high (55.8%). The proportion of obese subjects was 25.4% (**Table 1**). In terms of behaviour and lifestyle variables, the salty food consumption was relatively high (39.1%). Half of subjects were reported to be physically less active (49.9%) whereas only 3% of subject had a sufficient intake of fruit and vegetables (97% vs 3%). Almost a third of the subjects (31.5%) smoked while alcohol consumption was rare (3.8% of subjects). The carbonated drink consumption was also less frequent in this study (3.8%).

**Table 1:** Baseline characteristics of the subjects

Variables	Category	n (%)
**Demographic characteristics**		
Age (years)	≥60	65 925 (16.9)
	18-59	323 168 (83.1)
Gender	Male	154 563 (39.7)
	Female	234 530 (60.3)
Occupation status	Employed	161 802 (41.6)
	Unemployed	227 291 (58.4)
Education	Low	172 112 (44.2)
	Mid-high	216 981 (55.8)
BMI	Obese*	98 659 (25.4)
	Non-obese	290 434 (74.6)
**Behaviour and life style**		
Consumption of salty food	Frequent (≥3 x/week)	152 151 (39.1)
	Seldom (<3 x/week)	236 942 (60.9)
Physical activity**†**	Less active	194 038 (49.9)
	Active	195 055 (50.1)
Consumption of fruit & vegetables‡	Less	377 487 (97.0)
	Adequate	11 606 (3.0)
Smoking	Yes	122 475 (31.5)
	No	266 618 (68.5)
Alcohol consumption	Yes	14 696 (3.8)
	No	374 397 (96.2)
Carbonated drink consumption	Frequent (≥3x/week)	14 901 (3.8)
	Seldom (<3x/week)	374 192 (96.2)
**Comorbid condition**		
Hypertensive	Yes	158 805 (40.8)
	No	230 288 (59.2)
Heart disease	Yes	10 105 (2.6)
	No	378 988 (97.4)
Diabetes mellitus	Yes	13 016 (3.3)
	No	376 077 (96.7)
Hepatitis	Yes	1922 (0.5)
	No	387 171 (99.5)
Stroke	Yes	6752 (1.7)
	No	382 341 (98.3)
**Water supply and complication during pregnancy**		
Water supply§	Not clean	141 670 (36.4)
	Clean	247 423 (63.6)
Complication during pregnancy	Hypertension	2228 (0.9)
	Other than hypertension	194 809 (83.1)
	Without complication	37 493 (16)
	Total	234 530 (100)
**Distribution and accessibility to health care facilities**		
Hospital	Yes	359 560 (92.4)
	Distant¶	122 165 (34.0)
	Near	237 395 (66.0)
	No	29 533 (7.6)
Primary health care	Yes	382 299 (98.3)
	Distant¶	17 109 (4.4)
	Near	365 190 (95.6)
	No	6794 (1.7)

BMI – body mass index

*Obese if BMI>27 kg/m^2^.

†Active physical activity – regularly doing moderate or high (both) physical activity. Less active – not regularly.

‡Consumption of fruit & vegetables – adequate if more than 5 portion/d for 7 d in a week.

§Classification of water supply based on JMP WHO – UNICEF 2006.

¶Distant – takes more than 30 min to access the health care facility.

### The prevalence of CKD

Based on the history of impaired kidney function diagnosed by physician, the prevalence of CKD found in this study was 0.5% (2085/389 093).

### Important demographic findings

Along with this observation, we studied that hypertension was the most prevalent comorbidity (40.8%) in our population, while the proportion of other metabolic diseases was rather low (DM 3.3%, heart disease 2.6%, stroke 1.7%) (**Table 1**). Despite the high proportion of subjects diagnosed with hypertension, anti-hypertensive medication was prescribed to only 36.2% of subjects, of which only 21.7% reported to use this medication regularly. In this cohort, 0.5% of the subjects has been diagnosed with hepatitis in their medical history.

Other parameters that we observed in this study were the distribution of clean water supply, complications during pregnancy and accessibility to health care facilities. The majority of subjects had access to clean water (63.6%). Among pregnant subjects, hypertension was responsible for only a minority of pregnancy-related complications (0.9%). Hospitals and primary health care facilities were mostly available for all subjects (92.4% and 98.3%, respectively), although 34% lived at a distant location from the hospital.

### The key risk factors for CKD

The bivariate analysis of the variables and their distribution in subjects with and without CKD is shown in Table S1 in the **Online Supplementary Document**. From all parameters, we found age group >60 years old (OR = 1.97; 95% CI = 1.79-2.17; *P* < 0.001), male (OR = 1.45; 95% CI = 1.33-1.59; *P* < 0.001), low education (OR = 1.45; 95% CI = 1.35-1.61; *P* < 0.001], less physical activity (OR = 1.38; 95% CI = 1.27-1.51; *P* < 0.001), smoking habit (OR = 1.22; 95% CI = 1.12-1.34; *P* < 0.001), heart disease (OR = 5.4; 95% CI = 4.74-6.17; *P* < 0.001), diabetes (OR = 4.23; 95% CI = 3.71-4.82; *P* < 0.001), stroke (OR = 4.04; 95% CI = 3.39-4.81; *P* < 0.001), hypertensive (OR = 1.94; 95% CI = 1.78-2.11; *P* < 0.001), and hepatitis (OR = 4.31; 95% CI = 3.18-5.85; *P* < 0.001) were the risk factors for CKD.

Multiple logistic regression analysis was performed to determine the relationship between risk factors and CKD. The first step was to use a candidate selection model where variables were selected from bivariate analysis when *P-*value was less than 0.25. The variables with *P-*value >0.25 (consumption of fruit and vegetables, alcohol consumption, carbonated drink consumption, clean water supply, and access to primary health care) were removed. We performed further analysis to assess for confounders using multiple logistic analysis, and we excluded variables with *P-*value >0.05, including salty food consumption (*P* = 0.468), occupation status (*P* = 0.143), smoking habit (*P* = 0.110), and BMI (*P* = 0.089). Afterward, we performed an interaction test between variables that were considered to influence the development of CKD (ie, hypertensive*heart disease, hypertensive*stroke, hypertensive*DM, physical activity*DM, physical activity*hypertensive, education status* physical activity, DM*heart disease, stroke*heart disease, age*hypertensive, age*heart disease, age*DM). The interactions between those variables in relation to CKD were statistically significant for interaction between hypertensive*stroke, age*hypertensive, age*heart disease, and age*DM (*P* = 0.009; *P* < 0.001; *P* = 0.003; and *P* = 0.022; respectively).

The multiple logistic regression analysis demonstrated that physical inactivity (OR = 1.236; 95% CI = 1.128-1.354; *P* < 0.001), low educational status (OR = 1.307; 95% CI = 1.191-1.434; *P* < 0.001), male (OR = 1.527; 95% CI = 1.398-1.668; *P* < 0.001), stroke (OR = 1.916; 95% CI = 1.570-2.338; *P* < 0.001), heart disease (OR = 2.941; 95% CI = 2.356-3.671; *P* < 0.001), DM (OR = 2.462; 95% CI = 1.979-3.063; *P* < 0.001), hypertension*age (OR = 1.434; 95% CI = 1.161-1.772; *P* < 0.001), heart disease*age (OR = 1.421; 95% CI = 1.073-1.880; *P* = 0.014), and stroke*hypertension (OR = 1.677; 95% CI = 1.030-2.730; *P* = 0.038) were significantly related to CKD, whereas hepatitis was the strongest risk factors for CKD (OR = 3.406; 95% CI = 2.496-4.648; *P* < 0.001) (**Table 2**).

**Table 2:** Multiple logistic regression analysis for risk factors of CKD

Variables	Β	*P-*value	OR	95% CI
Physical inactivity	0.212	<0.001	1.236	1.128-1.354
Low educational status	0.268	<0.001	1.307	1.191-1.434
Older age (≥60 y)	-0.972	0.005	0.378	0.192-0.744
Hypertensive	-0.903	0.064	0.405	0.156-1.054
Male gender	0.423	<0.001	1.527	1.398-1.668
Hepatitis	1.225	<0.001	3.406	2.496-4.648
Stroke	0.650	<0.001	1.916	1.570-2.338
Heart disease	1.079	<0.001	2.941	2.356-3.671
Diabetes mellitus	0.901	<0.001	2.462	1.979-3.063
Access to hospital	-0.081	0.037	0.923	0.855-0.995
Hypertension*age	0.361	0.001	1.434	1.161-1.772
Heart disease*age	0.351	0.014	1.421	1.073-1.880
Diabetes*age	0.217	0.126	1.242	0.941-1.640
Stroke*hypertension	0.517	0.038	1.677	1.030-2.730

CI – confidence interval, OR – odds ratio

Subsequently, we further analysed the association between CKD and hepatitis positive subjects. Out of 1922 hepatitis positive participants, 43 subjects were diagnosed with CKD. Afterward, we analysed the proportion of each variable that were considered to influence the development of CKD in hepatitis-positive subjects. These include hypertension (46.5%), DM (20.9%), male sex (58.1%), and age <50 years (48.8%) (**Table 3**). The association of variables and CKD in hepatitis-positive subjects is shown in **Table 4**. DM was the only variable that was significantly associated with CKD in hepatitis-positive subjects in the bivariate analysis (OR = 4.34; 95% CI = 2.03-9.28; *P* < 0.001).

**Table 3:** Proportion of risk factors in CKD subjects with hepatitis

Variables	Category	n (%)
Hypertensive	Yes	20 (46.5)
	No	23 (53.5)
Diabetes mellitus	Yes	9 (20.9)
	No	34 (79.1)
Gender	Male	25 (58.1)
	Female	18 (41.9)
Age (years)	<50	21 (48.8)
	≥50	22 (51.2)

**Table 4:** Bivariate analysis on the association of risk factors and CKD in hepatitis-positive subjects

Variables	CKD				Total		OR (95% CI)	*P-*value
**Yes**		**No**	
**n**	**%**	**N**	**%**	**n**	**%**		
**Diabetes mellitus**								
Yes	9	7.7	108	92.3	117	100	4.34 (2.03-9.28)	<0.001
No	34	0.5	1771	98.1	1805	100		
Total	43	2.2	1879	97.8	1922	100		
**Hypertensive**								
Yes	20	2.5	770	97.5	790	100	1.25 (0.68-2.29)	0.531
No	23	2.0	1109	98.0	1132	100		
Total	43	2.2	1879	97.8	1922	100		
**Gender**								
Male	25	2.8	868	97.2	893	100	1.61 (0.87-2.98)	0.121
Female	18	1.7	1011	98.3	1029	100
Total	43	2.2	1879	97.8	1922	100		
**Age (years)**								
<50	21	1.7	1193	98.3	1214	100	0.54 (0.30-1.05)	0.053
≥50	22	3.1	686	96.9	708	100		
Total	43	2.2	1879	97.8	1922	100		

CI – confidence interval, CKD – chronic kidney disease, OR – odds ratio

In addition, using multiple logistic regression analysis, we selected DM and male gender to be included in the further analysis (*P-*value <0.25). In the multiple logistic regression analysis, we observed that DM (OR = 4.280; 95% CI = 3.756-4.876; *P* < 0.001) and male subjects (OR = 1.474; 95% CI = 1.352-1.606; *P* < 0.001) were identified as independent risk factors for CKD in hepatitis-positive subjects.

## DISCUSSION

In the present study we found that the prevalence of CKD was relatively low, with most population being young adults, along with a high rate of unemployment. Unexpectedly, hepatitis was a prominent risk factor for CKD. The prevalence of CKD was higher than the rate that has been published in the earlier report of the national survey (Riskesdas 2018) [4]. This observation was likely due to the subject selection method where we excluded children under 18 years old. Although we found a significant increase in the prevalence of CKD (ie, from 0.38% to 0.5%), this rate was lower than expected from previous studies [1,10-12]. In this survey, the diagnosis of CKD was established given the history of decreasing renal function made by physicians, not based on the eGFR measurement nor abnormalities found in urinalysis. Therefore, it might underestimate the actual number of CKD patients in our population. The majority of our population was young adult aged 18-59 with a high rate of unemployment. It might be due to most subjects participated in this survey being female and housemaids. Even though a large portion of our population has access to clean water, there was a substantial part without the access. Most subjects have access to health care facilities although more than one-third of them live distantly from the hospital. In the multiple logistic regression analysis we found that older age was not a risk factor for CKD, which is in contrast with previous studies [13,14]. In our cohort, it appears that older age becomes a protective factor for developing CKD. However, this survey captured only 16.9% of individuals aged sixty years or older which may contribute to the result, even though the proportion of CKD in this population was higher compared to younger subjects. Remarkably, hypertension appeared not to be associated with CKD in the multiple logistic regression analysis, whereas various other studies did observe that hypertension was positively associated with the risk of CKD [15-17]. We considered that this result might be influenced by the high number of missing subjects in the initial data cleaning (missing data on hypertension diagnosis was up to 40.5% of total initial recruitment data). Despite the fact that hypertension prevalence was high in our population, we found that only 36.2% of hypertensive subjects received anti-hypertensive medication of which a vast minority used this medication regularly (21.7%). This possibly indicates that the people’s awareness of the disease is low, paired with a poor understanding of the disease and treatment, and lack of facilities in the health care such as medication.

The rising prevalence of CKD is related to the increasing number of vulnerable subjects with metabolic condition such as hypertension, DM and pre-diabetes [14]. Despite the fact that hypertension was the major comorbidity presented in our study, the prevalence of other metabolic disorders such as DM, heart disease and stroke were relatively low. In this respect, it is important to notice that Indonesia is currently facing a demographic and epidemiological transition. Epidemiological transitions appeared in the shift in disease patterns and causes of death which were previously caused by infectious diseases or communicable diseases. Now these are commonly caused by chronic or non-communicable disease and degenerative diseases, and the challenge of re-emerging diseases such as tuberculosis and malaria [18-20]. In 2006, Prodjosudjadi et al. stated the leading cause of ESKD in patients who underwent haemodialysis in Indonesia was chronic glomerulonephritis (39.87%) followed by diabetic nephropathy (17.54%), hypertension (15.72%), obstructive and infectious diseases (13.44%), and polycystic kidney disease (2.51%) [21]. This study proved that traditional risk factors such as DM, heart disease, and stroke played an independent role as risk factors for CKD, although their prevalence was relatively lower than we expected. Following the original report of the national survey, we noticed that there was a rise in the prevalence of DM found in this study (ie, from 2.0% to 3.3%) as we only studied adult population (ie, ≥18 years old), excluding the group younger 18 years old analysed in the previous survey [4]. The diagnosis of DM was further improved in the national survey using the blood glucose measurement in approximately 37 460 subjects [4], and resulted in a higher prevalence of DM (ie, 10.9%) which corresponds to the results of previous studies [22-24]. This result suggests the burden of metabolic disease in our population is significantly increasing and this would probably influence the development of CKD in our population.

Hepatitis was found to be an important risk factor of CKD in this study. Viral hepatitis is a crucial public health drawback in our country, in particular hepatitis B and C which elicit the problems of chronicity. Based on a policy report of viral hepatitis situation in Indonesia (2018), it was estimated 19 million people were infected with hepatitis B virus (HBV) and 2.5 million people with hepatitis C virus (HCV) – and the death rate is increasing from both infections [25]. The prevalence of HBV infection in Indonesia was 9.4% in 2007 and has declined to 7.1% in 2013, indicating that Indonesia has shifted from high to moderate endemicity of HBV infection [26]. The incidence of hepatitis C was estimated at 0.05%-3.37% [25]. Among vulnerable groups such as haemodialysis patients, people who inject illicit drugs, and health care workers, the prevalence of viral hepatitis was found to be higher. Hepatitis C infection rates in haemodialysis patients ranges from 61%-83.2% and was independently correlated with dialysis vintage and the number of blood transfusions [25].

The type of renal involvement in HBV infection is wide, including membranous glomerulonephritis, membranoproliferative glomerulonephritis (MPGN), polyarteritis nodosa (PAN), mesangial proliferative glomerulonephritis, IgA nephropathy, amyloidosis, and serum-sickness-like syndrome [27,28]. When we look at Indonesian data in the histopathology review of kidney biopsies, membranous nephropathy, MPGN, and mesangial proliferative nephritis accounted for only 3.7%, 2.5%, and 3.1% of primary glomerular disease, respectively, while lupus nephritis was the most common finding among secondary glomerulonephritis (16.7%) [29]. Other specific HBV infection-associated renal pathology such as PAN or serum-sickness-like syndrome were not clearly captured [29]. It is important to study the clinical-histopathological relationship on the exact cause of the kidney disease in more detail in the future, in order to evaluate the influence of HBV infection on the prevalence of kidney diseases in Indonesia.

While the clinical spectrum of HCV-associated nephropathies are cryoglobulinemia, which accounted for the most common diseases such as membranous nephropathy, focal segmental glomerulosclerosis, IgA nephropathy, fibrillary and immunotactoid glomerulopathy [30]. A large cohort study in Taiwan, a country with a high prevalence of HCV infection, on the association of HCV infection and CKD indicated that the incidence of CKD was 2.0% after 6-year follow-up among HCV-infected subjects with no traditional CKD risk factors; the risk of developing CKD was significantly higher in HCV-infected group (adjusted hazard ratio = 1.75, 95% CI = 1.25-2.43, *P* = 0.0009) [31]. Large cohort studies have found that subjects with HCV infection have a higher risk and shorter time to develop CKD [32,33]. The higher risk of developing CKD was reported in younger age (<50 years), male gender, those with comorbid diabetes, hypertension, hyperlipidaemia, cirrhosis, genetic factor, and nephrotoxic agents [34]. Our study suggested that DM and male gender were the independent risk factor for CKD in hepatitis-positive subjects. This might also support the paradigm of epidemiological transition where communicable disease is remaining high in our population (ie, hepatitis), while the non-communicable disease event is growing (ie, DM).

Gender difference has been known to impact the CKD development, although conflicting data has been published [13]. In this study we found that male subjects have a higher risk of developing CKD. This result is parallel to a cross-sectional community-based study in Japan where they observed that among elderly subjects age >65 years, male gender is associated with CKD (OR = 2.97; 95% CI = 1.33-6.62) [16], while Lin et al. reported that women had a higher CKD prevalence than men (14.8% vs 12.5%, *P* = 0.005) across stages of CKD [15]. Nevertheless, kidney function declines happen more rapidly in men compared to women, possibly due to men having a worse lifestyle as well as the protective effects of oestrogen or the harmful effects of testosterone [35].

Physical inactivity was observed to be a risk factor of CKD in this survey. It might be linked to the other metabolic disorders such as obesity even though it has not been to be proven related to CKD.

This study confirmed that traditional risk factors together with communicable disease were the independent risk factors for CKD. A comprehensive CKD surveillance program targeting high-risk populations, including those with diabetes, stroke, heart disease, hepatitis, male, physical inactivity, and low education status is crucial for the early detection of CKD and ameliorating the burden of the disease in Indonesia. The CKD screening program involving measurement of eGFR or albuminuria/proteinuria, or both is well established [36,37]. Screening programs using albuminuria are widely recommended, especially for high-risk CKD population, even though a substantial number of individuals may be undetected (45%) when this method is only applied to the high-risk setting [38].

Our study has some limitations. First, the definition of CKD was made based on the diagnosis of declining kidney function made by physicians rather than directly measuring the eGFR or albuminuria. Consequently, patients at some stage of eGFR may have been incorrectly diagnosed as having no kidney disease. Besides, the diagnosis of hepatitis was also made based on the patient history. Lastly, the cross-sectional design of this study which prevents the establishment of temporality between risk factors and the incidence of CKD.

## CONCLUSIONS

This population-based survey confirmed the increasing burden of CKD in Indonesia and suggested that besides traditional metabolic risk factors, viral hepatitis has proven to be an independent risk factor for CKD in Indonesia. Furthermore, the risk of CKD is greater in male hepatitis patients with DM. The result of this study might be considered as support for the need for an aggressive screening program for patients with a high risk for the development of CKD. Apart from patients with traditional cardiometabolic risk factors, such a program should include patients with viral hepatitis.

## Additional material


Online Supplementary Document
