# Supplementary material for: Prevalence and risk factors for chronic kidney disease in Indonesia: An analysis of the National Basic Health Survey 2018
Source: J Glob Health. 2022 Oct 14;12:04074. doi: 10.7189/jogh.12.04074 (PMC9559178; doi:10.7189/jogh.12.04074)
Supplement: Online Supplementary Document [file jogh-12-04074-s001.pdf]

**ONLINE SUPPLEMENTARY DOCUMENT**

**Title: Prevalence and risk factors for chronic kidney disease in Indonesia: An analysis of the National Basic Health Survey 2018**

**Authors: Ni Made Hustrini, Endang Susalit, Joris I. Rotmans**

**Table S1. Bivariate analysis of proportion difference for CKD across variables**

| Variables | CKD | | | | Total | | OR (95% CI) | *P-value* |
|---|---|---|---|---|---|---|---|---|
| | Yes | | No | | | | | |
| | n | % | n | % | n | % | | |
| **Age group** | | | | | | | | |
| ≥60 years old | 597 | 0.9 | 65,328 | 99.1 | 65,925 | 100 | 1.97 (1.79 – 2.17) | <0.001 |
| 18-59 years old | 1,488 | 0.5 | 321,680 | 99.5 | 323,168 | 100 | | |
| Total | 2,085 | 0.5 | 387,008 | 99.5 | 389,093 | 100 | | |
| **Gender** | | | | | | | | |
| Male | 1,021 | 0.7 | 153,542 | 99.3 | 154,563 | 100 | 1.45 (1.33 – 1.59) | <0.001 |
| Female | 1,064 | 0.5 | 233,466 | 99.5 | 234,530 | 100 | | |
| Total | 2,085 | 0.5 | 387,008 | 99.5 | 389,093 | 100 | | |
| **Education** | | | | | | | | |
| Low | 1,125 | 0.7 | 170,987 | 99.3 | 172,112 | 100 | 1.48 (1.35 – 1.61) | <0.001 |
| Mid - high | 960 | 0.4 | 216,021 | 99.6 | 216,981 | 100 | | |
| Total | 2,085 | 0.5 | 387,008 | 99.5 | 389,093 | 100 | | |
| **Occupation** | | | | | | | | |
| Employed | 911 | 0.6 | 160,891 | 99.4 | 161,802 | 100 | 1.09 (1.00 – 1.18) | 0.053 |
| Unemployed | 1,174 | 0.5 | 226,117 | 99.5 | 227,291 | 100 | | |
| Total | 2,085 | 0.5 | 387,008 | 99.5 | 389,093 | 100 | | |
| **Consumption of fruit & vegetables** | | | | | | | | |
| Less | 2,022 | 0.5 | 375,465 | 99.5 | 377,487 | 100 | 0.98 (0.76 – 1.26) | 0.968 |
| Adequate‡ | 63 | 0.5 | 11,543 | 99.5 | 11,606 | 100 | | |
| Total | 2,085 | 0.5 | 387,008 | 99.5 | 389,093 | 100 | | |
| **Consumption of salty food** | | | | | | | | |
| Frequent | 762 | 0.5 | 151,389 | 99.5 | 152,151 | 100 | 0.89 (0.82 – 0.98) | 0.017 |
| Seldom | 1,323 | 0.6 | 235,619 | 99.4 | 236,942 | 100 | | |
| Total | 2,085 | 0.5 | 387,008 | 99.5 | 389,093 | 100 | | |
| **Physical activity** | | | | | | | | |

| | | | | | | | | |
|---|---|---|---|---|---|---|---|---|
| Less active | 1,208 | 0.6 | 192,830 | 99.4 | 194,038 | 100 | 1.38 (1.27 – 1.51) | <0.001 |
| Active† | 877 | 0.4 | 194,178 | 99.6 | 195,055 | 100 | | |
| Total | 2,085 | 0.5 | 387,008 | 99.5 | 389,093 | 100 | | |
| **Smoking** | | | | | | | | |
| Yes | 751 | 0.6 | 121,724 | 99.4 | 122,475 | 100 | 1.22 (1.12 – 1.34) | <0.001 |
| No | 1,334 | 0.5 | 265,284 | 99.5 | 266,618 | 100 | | |
| Total | 2,085 | 0.5 | 387,008 | 99.5 | 389,093 | 100 | | |
| **Alcohol consumption** | | | | | | | | |
| Yes | 71 | 0.5 | 14,625 | 99.5 | 14,696 | 100 | 0.89 (0.70 – 1.13) | 0.404 |
| No | 2,014 | 0.5 | 372,383 | 99.5 | 374,397 | 100 | | |
| Total | 2,085 | 0.5 | 387,008 | 99.5 | 389,093 | 100 | | |
| **Carbonated drink consumption** | | | | | | | | |
| Frequent | 89 | 0.6 | 14,812 | 99.4 | 14,901 | 100 | 1.12 (0.90 – 1.38) | 0.322 |
| Seldom | 1,996 | 0.5 | 372,196 | 99.5 | 374,192 | 100 | | |
| Total | 2,085 | 0.5 | 387,008 | 99.5 | 389,093 | 100 | | |
| **Heart disease** | | | | | | | | |
| Yes | 258 | 2.6 | 9,847 | 97.4 | 10,105 | 100 | 5.40 (4.74– 6.17) | <0.001 |
| No | 1,827 | 0.5 | 377,161 | 99.5 | 378,988 | 100 | | |
| Total | 2,085 | 0.5 | 387,008 | 99.5 | 389,093 | 100 | | |
| **Diabetes Mellitus** | | | | | | | | |
| Yes | 263 | 2 | 12,753 | 98 | 13,016 | 100 | 4.23 (3.71 – 4.82) | <0.001 |
| No | 1,822 | 0.5 | 374,255 | 99.5 | 376,077 | 100 | | |
| Total | 2,085 | 0.5 | 387,008 | 99.5 | 389,093 | 100 | | |
| **Stroke** | | | | | | | | |
| Yes | 137 | 2 | 6,615 | 98 | 6,752 | 100 | 4.04 (3.39 – 4.81) | <0.001 |
| No | 1,948 | 0.5 | 380,393 | 99.5 | 382,341 | 100 | | |
| Total | 2,085 | 0.5 | 387,008 | 99.5 | 389,093 | 100 | | |
| **Hypertensive** | | | | | | | | |
| Yes | 1,192 | 0.8 | 157,613 | 99.2 | 158,805 | 100 | 1.94 (1.78 – 2.11) | <0.001 |
| No | 893 | 0.4 | 229,395 | 99.6 | 230,288 | 100 | | |
| Total | 2,085 | 0.5 | 387,008 | 99.5 | 389,093 | 100 | | |
| **Hepatitis** | | | | | | | | |
| Yes | 43 | 2.2 | 1,879 | 97.8 | 1,922 | 100 | 4.31 (3.18 – 5.85) | <0.001 |
| No | 2,042 | 0.5 | 385,129 | 99.5 | 387,171 | 100 | | |

| | | | | | | | | |
|---|---|---|---|---|---|---|---|---|
| Total | 2,085 | 0.5 | 387,008 | 99.5 | 389,093 | 100 | | |
| **Body mass index** | | | | | | | | |
| Obese* | 567 | 0.6 | 98,092 | 99.4 | 98,659 | 100 | 1.10 (0.99 – 1.21) | 0.056 |
| Non-Obese | 1,518 | 0.5 | 288,916 | 99.5 | 290,434 | 100 | | |
| Total | 2,085 | 0.5 | 387,008 | 99.5 | 389,093 | 100 | | |
| **Complication during pregnancy** | | | | | | | | |
| Hypertension | 11 | 0.5 | 2,217 | 99.5 | 2,228 | 100 | 0.42 (0.22 – 0.80) | 0.008 |
| Other than hypertension | 974 | 0.5 | 193,835 | 99.5 | 194,809 | 100 | 0.39 (0.33 – 0.52) | <0.001 |
| Without complication | 79 | 0.2 | 37,414 | 99.8 | 37,493 | 100 | | |
| Total | 1,064 | 0.5 | 233,466 | 99.5 | 234,530 | 100 | | |
| **Water supply§** | | | | | | | | |
| Not clean | 770 | 0.5 | 140,900 | 99.5 | 141,670 | 100 | 1.02 (0.93 – 1.11) | 0.637 |
| Clean | 1,315 | 0.5 | 246,108 | 99.5 | 247,423 | 100 | | |
| Total | 2,085 | 0.5 | 387,008 | 99.5 | 389,093 | 100 | | |
| **Access to hospital** | | | | | | | | |
| None | 37 | 0.5 | 6,757 | 99.5 | 6,794 | 100 | 1.128 (0.941-1.351) | 0.193 |
| Yes, near access | 1964 | 0.5 | 363,226 | 99.5 | 365,190 | 100 | 1.071 (0.974-1.178) | 0.158 |
| Yes, distant access | 84 | 0.5 | 17,025 | 99.5 | 17,109 | 100 | | |
| Total | 2,085 | 0.5 | 387,008 | 99.5 | 389,093 | 100 | | |
| **Access to primary health care** | | | | | | | | |
| Yes, distant access¶ | 684 | 0.6 | 121,481 | 99.5 | 121,481 | 100 | 0.857 (0.579-1.268) | 0.440 |
| Yes, near access | 1254 | 0.5 | 236,141 | 99.5 | 237,395 | 100 | 0.893 (0.714-1.118) | 0.324 |
| None | 147 | 0.5 | 29,386 | 99,5 | 29,533 | 100 | | |
| Total | 1,838 | 0.5 | 354,155 | 99.5 | 355,993 | 100 | | |

*Obese if BMI >27 kg/m$^2$
†Active physical activity: regularly doing moderate or high (both) physical activity. Less active: not regularly.
‡Consumption of fruit & vegetables: adequate if more than 5 portion/day for 7 days in a week.
§Classification of water supply based on JMP WHO – UNICEF 2006.
¶Distant: takes more than 30 minutes to access the healthcare facility.
BMI – body mass index
